# The impact of diet and sociodemographic factors on cardiovascular health among students at Chimborazo polytechnic high school and State University of Milagro

**DOI:** 10.3389/fpubh.2025.1514146

**Published:** 2025-09-01

**Authors:** Verónica Patricia Sandoval-Tamayo, Angélica María Solís-Manzano, María Victoria Padilla-Samaniego, Katherine Denisse Suárez-González, Edgar Rolando Morales-Caluña, Dennis Alfredo Peralta-Gamboa

**Affiliations:** ^1^State University of Milagro, Faculty of Health Sciences, Milagro, Ecuador; ^2^Faculty of Postgraduate, State University of Milagro, Milagro, Ecuador

**Keywords:** balanced diet, sociodemographic factors, cardiovascular health, university students, triglyceride levels, blood pressure

## Abstract

**Introduction:**

Cardiovascular diseases (CVDs) represent a significant global public health burden, accounting for approximately 31% of annual deaths worldwide. Diet quality and sociodemographic factors are recognized as critical determinants influencing cardiovascular risk, particularly among young adults. University students are considered a vulnerable population due to rapid lifestyle transitions that may affect long-term health outcomes. This study aimed to analyze the associations between dietary patterns, sociodemographic factors, and cardiovascular health indicators in Ecuadorian university students.

**Methods:**

A cross-sectional study was conducted between 2022 and 2023 among 404 students from the State University of Milagro (UNEMI) and the Polytechnic School of Chimborazo (ESPOCH). Data on sociodemographic variables, dietary intake, and cardiovascular biomarkers were collected using structured interviews, clinical measurements, and blood analyses. Dietary intake was assessed using a 24-hour recall and a food frequency questionnaire adapted from the Block Screening Questionnaire. Cardiovascular health indicators included triglycerides, LDL and HDL cholesterol, systolic and diastolic blood pressure, and glucose levels. Associations between diet, sociodemographic factors, and cardiovascular outcomes were analyzed using one-way ANOVA, stratified by sex, with effect size estimation (η²) and Bonferroni corrections for multiple comparisons.

**Results:**

Among the participants (58.3% women, 41.7% men, mean age = 23.3 ± 4.4 years), 22% reported a very high-fat diet, whereas only 2% followed a nutrient-rich diet. Women with a very high-fat diet exhibited significantly higher triglyceride levels (mean = 189.6 mg/dL) compared to those with a low-fat diet (mean = 132.4 mg/dL, *p* = 0.010). Men with high-fat diets showed elevated systolic (mean = 128.3 mmHg) and diastolic blood pressure (mean = 83.7 mmHg) compared to those with low-fat diets (*p* < 0.01). A nutrient-rich diet was associated with lower systolic blood pressure in women (mean = 106.5 mmHg vs. 113.7 mmHg, *p* < 0.001). Place of birth influenced cardiovascular outcomes: men from the Highlands had higher LDL cholesterol (145.2 mg/dL vs. 126.3 mg/dL, *p* = 0.005), while women born in the Coastal region showed lower systolic blood pressure (108.6 mmHg vs. 118.2 mmHg, *p* = 0.004). Place of residence was associated with systolic pressure in men (*p* = 0.021), and father’s origin significantly affected LDL and diastolic values in men (*p* < 0.01).

**Discussion and conclusions:**

This study demonstrates significant associations between dietary quality, sociodemographic background, and cardiovascular health among Ecuadorian university students. High-fat diets were linked to adverse lipid profiles and increased blood pressure, while nutrient-rich diets showed protective effects, especially among women. Regional and familial origins influenced cardiovascular risk markers, underscoring the relevance of socioeconomic and environmental determinants. Public health strategies should target dietary education, nutritional interventions, and region-specific policies to mitigate cardiovascular risk in young adult populations. Future research should adopt longitudinal and multivariable models to strengthen causal inferences and validate observed associations.

## Introduction

Cardiovascular diseases continue to be an issue of global relevance representing an alarming indicator in public health due to their high prevalence and persistent consequences (1), which have a significant influence on the “mortality” of the population (2) consider that “each year, 17.9 million people die from cardiovascular diseases, accounting for approximately 31% of all deaths worldwide” as a striking illustration of the significant burden.

In recent years, an increase in the prevalence of cardiovascular diseases has been observed, especially among young adults due to a decrease in diet quality which is related to an increase in the risk of acquiring cardiovascular diseases including coronary artery disease (3), “central obesity, abnormal blood glucose levels and abnormal body mass index (BMI)” leading to stroke (4). As indicated by (5) among the multiple influencing “cardiovascular risk factors” is diet, including dietary habits that are determinants in the onset and progression of diseases such as hypertension, dyslipidemia, and obesity, according to (6), research indicates that replacing animal proteins with vegetable proteins, carbohydrates with vegetable proteins, or carbohydrates with monounsaturated fats and proteins is associated with a decrease in the mortality rate due to cardiovascular diseases.

In addition, several studies have shown that socio-demographic factors—such as socioeconomic status, place of residence, and access to food resources—can significantly influence dietary patterns, thereby affecting cardiovascular health (7), In line with this, the present study examines socio-demographic factors including place of birth, place of usual residence, and parental origin, and how they relate to cardiovascular health indicators.

University students constitute a population of particular interest. During this transitional life stage into adulthood, students often experience significant changes in their lifestyle and eating habits, which can directly impact their long-term cardiovascular health. This is a critical period to identify early risk patterns and promote healthy behaviors that persist over time. Previous research has documented that university students tend to have poor dietary habits, including low consumption of fiber, fruits, and vegetables, combined with high intake of fat. Additionally, a sedentary lifestyle, tobacco use, and alcohol consumption are common in this group, all of which are recognized cardiovascular risk factors (8).

Despite the global burden of cardiovascular diseases (CVDs), which are the leading cause of death worldwide, there is a growing concern about their prevalence among young adults, including university students. Locally, in Ecuador, this issue is especially pressing due to dietary transitions and regional inequalities in access to healthcare and nutritious food. The cities of Milagro and Riobamba, where this study was conducted, represent contrasting socioeconomic environments—urban vs. semi-urban settings—with differing public health challenges. Understanding how these local contexts influence cardiovascular health can inform tailored interventions for at-risk populations.

In this context, the present study aimed to analyze the association between diet and sociodemographic factors on cardiovascular health among students from two public universities in Ecuador: the State University of Milagro (UNEMI) and the Polytechnic School of Chimborazo (ESPOCH).

## Materials and methods

### Sample selection, inclusion and exclusion criteria

A total of 404 university students from the State University of Milagro (UNEMI) and the Polytechnic School of Chimborazo (ESPOCH) were selected using a stratified random sampling method. Inclusion criteria included students aged between 18 and 55 years, currently enrolled in undergraduate programs at the time of data collection, and who voluntarily agreed to participate in the study. Exclusion criteria included individuals with a prior diagnosis of cardiovascular disease or chronic illness that could confound cardiovascular biomarkers, and those who did not complete the dietary or clinical assessments.

### Study setting

The study involved students from two distinct regions of Ecuador: the Coastal and Andean highland areas. Participants from the State University of Milagro (UNEMI) are predominantly from Milagro, a mid-sized city located in the Coastal region of Ecuador, characterized by a warm tropical climate, low-altitude geography, and an economy centered on agriculture, agro-industry, and commerce. In contrast, students from the Polytechnic School of Chimborazo (ESPOCH) are primarily from Riobamba and surrounding areas in the Andean Sierra region, situated at a much higher altitude with a temperate to cold climate. This region’s economy is more rural and based on agriculture, small-scale trade, and public services, with many communities facing geographic and economic barriers to accessing health services and nutritional resources.

In this study, income level classifications were operationalized based on regional economic activity, access to public infrastructure, and urbanization. The Coastal region, particularly Guayaquil and its surrounding cities like Milagro, was categorized as a higher-income area due to its concentration of industrial and commercial activity, higher availability of healthcare services, and greater connectivity. Meanwhile, the Sierra region, particularly rural areas surrounding Riobamba, was categorized as a lower-income area, defined by lower household income averages, reduced access to specialized healthcare, and greater prevalence of food insecurity and poverty. These contextual differences may partly explain the disparities observed in cardiovascular health indicators across the two student populations.

### Sample size calculation

The sample size was calculated using a confidence level of 95%, a 5% margin of error, and a population size of approximately 8,000 students from the two universities. Based on these parameters, the minimum sample size required was 367. A total of 404 students participated, ensuring the representativeness of the study and accounting for potential non-responses or data inconsistencies.

### Ethical considerations and data protection

This study was conducted in accordance with the Declaration of Helsinki. All procedures involving human participants were approved by the Ethics Committee in Human Research of the Polytechnic School of Chimborazo (ethics approval number IO-07-CEISH-ESPOCH-2023). Written informed consent was obtained from all participants. To ensure confidentiality, data were anonymized and stored securely in encrypted databases accessible only to the research team.

### Data collection procedures

Sociodemographic and dietary data were obtained through structured interviews. Clinical data were gathered by qualified personnel at university health centers. Anthropometric and biochemical assessments included blood pressure, oxygen saturation, and blood analysis for lipid profiles, glycemia, and cholesterol levels.

### Dietary assessment

Dietary intake was evaluated using a 24-h dietary recall and a food frequency questionnaire adapted from the Block Screening Questionnaire (9). Macronutrient and micronutrient intake (iron, calcium, vitamin C) were estimated using the FAO/INFOODS regional food composition tables.

Diet classification:

- Fat intake: Categorized into very high (\u226550 points), high (40–49), moderate (30–39), low (20–29), and very low (<20).
- Nutrient adequacy was categorized into three levels: nutrient-rich diets met more than 80% of daily recommendations for at least three key nutrients; nutrient-sufficient diets met between 50 and 80% of the recommendations; and nutrient-poor diets met less than 50% of the recommended intake.

### Data analysis and statistical tests

While the study acknowledges the analytical limitations of relying on bivariate analyses such as one-way ANOVA, the choice not to apply multivariable regression models was primarily based on the exploratory nature of the research and the need to ensure interpretability in a sample of limited size. Although key covariates such as age, sex, BMI, and region of residence were collected, the study prioritized stratified analyses and effect size estimation (e.g., η^2^) to highlight subgroup differences. Nevertheless, future research should incorporate multivariable linear regression models to adjust for confounding factors and better isolate the specific impact of dietary patterns on cardiovascular outcomes.

In terms of dietary assessment, the adapted questionnaire was based on the Block Screening Questionnaire for Fat, Fruit, and Fiber Intake (9), a tool with established construct validity in previous international applications. The current study applied a translated and regionally adapted version, and although internal reliability coefficients (e.g., Cronbach’s alpha) were not formally computed, content validity was preserved through expert review by registered dietitians and public health researchers familiar with the Ecuadorian context.

The classification of dietary adequacy—specifically, nutrient-rich, sufficient, and poor diets—was informed by thresholds aligned with FAO/INFOODS guidelines and recommendations from the World Health Organization (WHO) and American Heart Association (AHA), particularly regarding minimum micronutrient intake levels. Nonetheless, the study recognizes the need for future validation studies in the Ecuadorian population to further refine these thresholds and improve external comparability.

Interaction effects between sex and diet were explored descriptively, and while no formal interaction tests were conducted in this version of the study, subgroup analyses by sex were performed to highlight relevant sex-specific patterns. Future work will consider testing statistical interactions to confirm differential effects across demographic groups.

### Measures of association and confounder adjustments

Effect sizes (e.g., Eta squared – η^2^) were calculated to assess the magnitude of differences observed in ANOVA tests. Analyses were stratified by sex to account for sex-based physiological differences. Although no multivariable regression models were applied, the influence of potential confounding variables is acknowledged and discussed as a limitation of the study.

### Health characteristics

Oxygen saturation was measured with a pulse oximeter and blood pressure with a digital upper arm blood pressure monitor. In addition, qualified laboratory personnel took venous blood samples for blood biometry and blood chemistry (total cholesterol, High-Density Lipoprotein (HDL), Low-Density Lipoprotein (LDL), triglycerides, and glucose).

### Characteristics of the diet

For the measurement of food consumption, a quantitative method (24-h recall) and a qualitative method (frequency of consumption by specific foods) were applied, the percentages of energy, macronutrient, and micronutrient adequacy (iron, calcium, vitamin C) were calculated, and food consumption frequencies were determined.

The dietary assessment was performed following the block screening questionnaire for fat, fruit, vegetable, and fiber intake, according to this block screening, scores for fat intake (fat points) and scores for fruit intake (fruit, vegetable, and fiber points) were established (9).

## Results

### Statistical characteristics of the sample

The sample consisted of 404 university students, with a gender distribution of 41.67% male and 58.33% female (Table 1). The mean age was 23.34 years (SD = 4.38), with a median of 22 years and an age range of 19 to 55 years.

**Table 1:** Sociodemographic characteristics of the participants.

Variable	Total (*n* = 404)	Men (*n* = 168)	Women (*n* = 236)
Age (mean ± SD)	23.34 ± 4.40	23.70 ± 4.50	23.10 ± 4.30
Age range	19–55	-	-
Place of birth - Coastal region	180 (44.60%)	80 (47.60%)	100 (42.40%)
Place of birth - Highland region	200 (49.50%)	78 (46.40%)	122 (51.70%)
Place of birth - Other	24 (5.90%)	10 (6%)	14 (5.90%)
Current residence - Milagro	220 (54.50%)	100 (59.50%)	120 (50.80%)
Current residence - Riobamba	160 (39.60%)	60 (35.70%)	100 (42.40%)
Current residence - Other	24 (5.90%)	8 (4.80%)	16 (6.80%)
Father’s place of origin - Coastal region	190 (47%)	90 (53.60%)	100 (42.40%)
Father’s place of origin - Highland region	190 (47%)	70 (41.70%)	120 (50.80%)
Father’s place of origin - Other	24 (5.90%)	8 (4.80%)	16 (6.80%)
Mother’s place of origin - Coastal region	200 (49.50%)	85 (50.60%)	115 (48.70%)
Mother’s place of origin - Highland region	180 (44.60%)	75 (44.60%)	105 (44.50%)
Mother’s place of origin - Other	24 (5.90%)	8 (4.80%)	16 (6.80%)

Regarding dietary fat intake, 22% of participants had a very high fat intake, 7% a high intake, 16% a moderate-fat diet, 26% a low-fat diet, and 29% followed an almost fat-free diet. Concerning fruit, vegetable, and fiber consumption, only 2% followed a nutrient-rich diet, while over 78% maintained a diet low in nutrient content.

### Results of statistical analysis

All statistical comparisons were complemented with 95% confidence intervals (CIs) to provide a more accurate estimation of the observed effects. Additionally, effect size measures (Eta squared, η^2^) were reported for each ANOVA to evaluate the magnitude of associations, beyond statistical significance. The number of observations (N) for each subgroup is explicitly stated in the results tables to ensure transparency and reproducibility.

Given the multiple subgroup comparisons conducted (by sex, dietary classification, and sociodemographic variables), we applied the Bonferroni correction in post-hoc analyses to reduce the risk of Type I error inflation. These adjusted comparisons are indicated where relevant. Tables were also reorganized to improve readability, eliminate redundant values, and present results in a more streamlined format.

### Diet and cardiovascular health

Table 2 summarizes the results of the one-way ANOVA assessing the relationship between dietary factors (fat intake and fruit, vegetable, and fiber scores) and cardiovascular health indicators. Among women, significant differences in triglyceride levels were observed based on fat intake (ANOVA, *F*(4, 230) = 3.50, *p* = 0.010). Women with a very high-fat diet had a mean triglyceride level of 189.6 mg/dL (SD = 35.20), compared to 132.40 mg/dL (SD = 28.70) in those with a low-fat diet.

**Table 2:** Results of the one-way analysis of variance between diet and cardiovascular health (*p* < 0.05).

Group	Cardiovascular indicator	Mean (SD)	*N*	*F*-statistic	*p*-value
Women - Very High Fat Intake	Triglycerides (mg/dL)	189.6 (35.20)	236	3.50	0.010
Women - Low Fat Intake	Triglycerides (mg/dL)	132.4 (28.70)	236	3.50	0.010
Men - High Fat Intake	Systolic Blood Pressure (mmHg)	128.30 (9.20)	168	3.80	0.007
Men - Low Fat Intake	Systolic Blood Pressure (mmHg)	117.40 (7.80)	168	3.80	0.007
Men - High Fat Intake	Diastolic Blood Pressure (mmHg)	83.70 (8.60)	168	4.17	0.004
Men - Low Fat Intake	Diastolic Blood Pressure (mmHg)	76.20 (7.40)	168	4.17	0.004
Women - Nutrient-Rich Diet	Systolic Blood Pressure (mmHg)	106.5 (7.1)	236	19.30	0
Women - Nutrient-Poor Diet	Systolic Blood Pressure (mmHg)	113.7 (9.3)	236	19.30	0

These differences are not only statistically significant but also clinically meaningful. Women with a very high-fat diet had a mean triglyceride level of 189.6 mg/dL, which surpasses the threshold of 150 mg/dL established by the American Heart Association (AHA) and the European Society of Cardiology (ESC) as indicative of borderline or high atherogenic risk. In contrast, the mean triglyceride level of 132.4 mg/dL in women with low-fat diets remains within the acceptable range. The absolute difference of 57.2 mg/dL between these two groups represents a 43.2% increase in triglyceride concentration, suggesting a substantial elevation in cardiovascular risk. This clinical interpretation reinforces the relevance of dietary patterns in modulating lipid profiles among young women. Future studies should consider longitudinal monitoring to determine whether such elevations persist and translate into long-term cardiovascular outcomes.

In men, systolic blood pressure was significantly higher among those consuming a high-fat diet (mean = 128.30 mmHg, SD = 9.20) compared to those on a low-fat diet (mean = 117.40 mmHg, SD = 7.80) (*F*(4, 164) = 3.80, *p* = 0.007).

Diastolic blood pressure also showed significant differences in men (*F*(4, 164) = 4.17, *p* = 0.004), being higher in those with a very high-fat diet (mean = 83.70 mmHg, SD = 8.60) compared to those with a low-fat diet (mean = 76.20 mmHg, SD = 7.40).

Among women, those with a nutrient-rich diet exhibited lower systolic blood pressure (mean = 106.50 mmHg, SD = 7.10) than those needing dietary supplementation (mean = 113.70 mmHg, SD = 9.30) (*F*(2, 23) = 19.30, *p* = 0).

### Sociodemographic factors and cardiovascular health

Tables 3, 4 display the associations between sociodemographic factors, parental origin, and cardiovascular health indicators. Place of birth was significantly associated with LDL cholesterol levels in men (*F* (2, 166) = 5.70, *p* = 0.005). Men born in Highlands region had a mean LDL level of 145.20 mg/dL (SD = 25.40), compared to 126.30 mg/dL (SD = 22.80) in those born in Coastal region.

**Table 3:** Results of the one-way analysis of variance between participants’ socio-demographic factors and cardiovascular health (*p* < 0.05).

Group	Cardiovascular indicator	Mean (SD)	*N*	*F*-statistic	*p*-value
Men - Born in Highlands	LDL Cholesterol (mg/dL)	145.20 (25.40)	168	5.70	0.005
Men - Born in Coastal	LDL Cholesterol (mg/dL)	126.30 (22.80)	168	5.70	0.005
Women - Born in Highlands	Systolic Blood Pressure (mmHg)	118.20	236	5.69	0.004
Women - Born in Coastal	Systolic Blood Pressure (mmHg)	108.60	236	5.69	0.004
Men - Living in Highlands	Systolic Blood Pressure (mmHg)	115.80	168	4.03	0.021
Men - Living in Coastal	Systolic Blood Pressure (mmHg)	124.30	168	4.03	0.021

**Table 4:** Result of one-way analysis of variance between socio-demographic factors of participants’ parents and cardiovascular health (*p* < 0.05).

Group	Cardiovascular indicator	Cardiovascular indicator	Mean (SD)	*N*	*F*-statistic	*p*-value
Men - Father from Highlands	LDL Cholesterol (mg/dL)	LDL Cholesterol (mg/dL)	148.70 (24.90)	168	5,43	0,006
Men - Father from Coastal	LDL Cholesterol (mg/dL)	LDL Cholesterol (mg/dL)	130.40 (23.20)	168	5,43	0,006

In women, systolic blood pressure varied significantly by place of birth (*F*(2, 232) = 5.69, *p* = 0.004), being lower in those born in Coastal region (mean = 108.60 mmHg) compared to those born in Highlands region (mean = 118.20 mmHg).

Place of residence was significantly associated with systolic blood pressure in men (*F*(2, 166) = 4.03, *p* = 0.021). Men living in Highlands region showed lower systolic blood pressure than those from Coastal region (Highlands region = 115.80 mmHg, Coastal region = 124.30 mmHg).

### Parental background and cardiovascular health

The father’s place of origin significantly affected LDL cholesterol levels (*F*(2, 166) = 5.43, *p* = 0.006) and diastolic pressure (*F*(2, 166) = 4.81, *p* = 0.011) in men. Participants whose fathers were from Highlands region had higher LDL cholesterol (mean = 148.70 mg/dL, SD = 24.90) compared to those whose fathers were from Coastal region (mean = 130.40 mg/dL, SD = 23.20). Diastolic blood pressure was also higher in this group (mean = 84.50 mmHg, SD = 8.30 vs. 76.90 mmHg, SD = 7.10).

The mother’s place of origin did not show statistically significant associations with cardiovascular indicators in women, although similar trends were observed.

These findings support the significant influence of dietary and sociodemographic factors on cardiovascular health indicators in university students, with clear differences by sex, dietary patterns, and geographic origin.

## Discussion

### Effects of diet on lipids and blood pressure

This study revealed statistically and clinically significant associations between dietary quality and cardiovascular biomarkers among university students, with notable sex-specific differences. Women consuming very high-fat diets exhibited elevated triglyceride levels (mean = 189.6 mg/dL), which exceed the threshold of 150 mg/dL defined by the American Heart Association (AHA) as indicative of increased atherogenic risk. This aligns with findings from Gao et al. (10), who reported strong links between high-energy, low-fiber diets and cardiovascular disease. Similarly, Lee et al. (11) observed that high triglyceride levels in women were associated with fat-heavy diets, particularly those lacking in fiber and plant-based nutrients. The absolute difference of 57.2 mg/dL between low-and high-fat diet groups in our sample (a 43.2% increase) suggests substantial biological impact, reinforcing the role of diet as a modifiable cardiovascular risk factor in young women.

In men, high-fat intake was associated with significantly elevated systolic (mean = 128.3 mmHg) and diastolic (mean = 83.7 mmHg) blood pressure levels. These findings are consistent with Tapsell et al. (12), who concluded that diets rich in saturated fats and low in whole grains, fruits, and vegetables contribute to increased cardiovascular risk. Our data also revealed that women consuming nutrient-rich diets had lower systolic pressure (mean = 106.5 mmHg) compared to those with nutrient-poor patterns (mean = 113.7 mmHg). This supports the protective role of micronutrients and fiber in vascular function, highlighting the importance of comprehensive dietary interventions within university populations.

### Effects of sociodemographic variables

Beyond dietary intake, the study identified significant associations between sociodemographic variables and cardiovascular outcomes. Men born in the Highland region had higher LDL cholesterol levels than those born in the Coastal region, while women from the Highlands exhibited higher systolic blood pressure. These findings may reflect disparities in early-life nutrition, environmental exposures, and healthcare access. Students from urbanized areas like Guayaquil (Coastal region) may benefit from more diverse diets and better health services compared to those from semi-urban or rural areas like Riobamba.

Parental origin also showed notable associations. Male students whose fathers were from the Highlands displayed higher LDL and diastolic pressure values than those whose fathers originated from the Coast. These intergenerational differences may stem from inherited behavioral patterns, long-term dietary habits, or epigenetic influences shaped by regional contexts. Similar conclusions have been reached in studies by Zhou et al. (13) and Bockarie et al. (14), which emphasize the importance of social determinants in shaping cardiometabolic risk from early life stages.

Prior studies have shown that socioeconomic status and place of origin significantly shape cardiovascular risk profiles, particularly through mediators such as dietary quality and access to health-promoting environments (15).

Moreover, the university setting acts as a dietary ecosystem that influences students’ eating behaviors. Economic constraints, time pressure, and limited access to nutritious options on campus contribute to the persistence of unhealthy eating habits. Smaller served portions change perceived norms about appropriate intake (22), and environmental cues like portion sizes and modeled behaviors in social contexts have been shown to shape norms around eating (23).

Although regional differences between the Coastal and Highland zones were explored descriptively through stratified analyses, the study did not include formal modeling of interactions between region of residence and dietary patterns. This limits the ability to detect cross-level influences where local food environments, economic infrastructure, and cultural dietary norms may moderate the relationship between diet and cardiovascular outcomes. The exclusion of interaction terms was due in part to sample size constraints and the exploratory scope of the study. However, future research should incorporate regression models with cross-interactions (e.g., region*diet quality) to examine whether the impact of dietary intake varies by geographic context. Such analyses would enhance the identification of territorial patterns and inform the design of region-specific interventions to address nutritional disparities and cardiovascular risk.

### Biological interpretation and public policy implications

The associations observed are supported by established pathophysiological mechanisms. Diets rich in saturated fats promote low-grade inflammation, oxidative stress, and endothelial dysfunction—key processes in the development of hypertension and dyslipidemia. Specifically, the release of pro-inflammatory cytokines and the reduction of nitric oxide availability impair vascular tone and lipid metabolism (24). Conversely, nutrient-rich diets offer anti-inflammatory and antioxidant benefits, enhancing vascular health.

Sex-specific responses may also stem from physiological differences. In women, estrogen modulates lipid metabolism by regulating hepatic lipase activity and influencing triglyceride storage and clearance. Women also have higher subcutaneous fat, which differs metabolically from visceral fat more common in men. In men, visceral adiposity is associated with sympathetic overactivity and increased peripheral resistance, leading to elevated diastolic pressure. These distinctions underline the importance of considering sex not merely as a confounder, but as a biological modifier in cardiovascular research.

Furthermore, while the study stratified analyses by sex and observed distinct associations—such as elevated triglyceride levels in women and increased diastolic blood pressure in men—it did not include formal interaction tests (e.g., sex*diet) in the statistical modeling. Future research should incorporate such interaction terms to validate whether these patterns are statistically different between sexes.

From a physiological standpoint, sex-specific differences in cardiovascular response to dietary intake are well-documented in the literature. In women, elevated triglyceride levels may be partly explained by hormonal influences such as estrogen fluctuations, which modulate lipid metabolism and influence hepatic lipase activity (16, 17). Women also tend to have a higher proportion of subcutaneous fat, which differs in metabolic behavior from the visceral fat more common in men (18, 19). Conversely, in men, the greater prevalence of visceral adiposity has been associated with increased vascular resistance and sympathetic activity, contributing to elevated diastolic blood pressure (20, 21). These biological distinctions highlight the importance of considering sex not only as a control variable but also as a potential effect modifier in cardiovascular research.

From a public health perspective, the findings underscore the need for territorially and demographically tailored interventions within university settings. In the Coastal region—where students showed lower systolic pressure but higher fat intake—strategies could include reformulating university cafeteria menus to limit saturated fat and introducing visual food labeling systems. In contrast, for students from the Highland region, who exhibited higher LDL cholesterol and blood pressure, interventions should prioritize increasing access to fresh produce and subsidizing nutrient-rich food options on or near campus.

Given the observed sex-specific associations—such as elevated triglycerides in women and higher diastolic pressure in men—nutrition education programs should also be stratified by sex. For example, workshops for female students could focus on managing lipid intake and increasing fiber-rich food consumption, while programs for male students might emphasize sodium reduction and blood pressure regulation.

These evidence-based strategies go beyond general recommendations and align more closely with the environmental, biological, and sociodemographic realities highlighted in this study. Universities should work collaboratively with public health authorities to develop policies that reflect these differentiated needs and reduce cardiovascular risk in vulnerable student subpopulations.

### Limitations

This study is cross-sectional in nature, limiting causal inference. Potential confounders such as physical activity, sleep quality, and stress were not measured, which may affect the observed associations. Dietary data relied on self-reported instruments, introducing the risk of recall and social desirability bias. Although validated tools were used, future research should include multivariate regression models and formal interaction testing to better isolate the effects of diet and sociodemographic variables.

## Conclusion

This study identified significant associations between dietary quality, sociodemographic factors, and cardiovascular health indicators among university students from two public institutions in Ecuador. High-fat diets were correlated with elevated triglyceride levels in women and increased blood pressure in men, while nutrient-rich diets were associated with lower systolic blood pressure in women. Geographic and familial origins also appeared to influence cardiovascular outcomes, particularly among male participants.

However, due to the cross-sectional nature of the research design, these findings should be interpreted as correlational rather than causal. The study does not allow for conclusions about temporal precedence or directionality, and unmeasured confounding variables—such as physical activity, sleep quality, or psychological stress—may have influenced the associations observed.

Despite these limitations, the results provide valuable insights for public health decision-makers, particularly in low-and middle-income countries undergoing dietary and lifestyle transitions. Efforts to promote cardiovascular health among young adults should consider both nutritional interventions—such as increasing the availability of fruits, vegetables, and healthy fats within university food environments—and regional disparities in access to resources. Universities may play a proactive role in fostering healthier habits by incorporating nutrition education, accessible dining options, and awareness campaigns into campus life.

Future research should adopt longitudinal or experimental designs to explore the underlying mechanisms linking diet, sociodemographic background, and cardiovascular risk. Such studies could validate and expand upon the present findings, evaluate the effectiveness of tailored interventions, and better inform evidence-based policies aimed at reducing long-term cardiovascular disease burden among young populations.

## Data Availability

The raw data supporting the conclusions of this article will be made available by the authors, without undue reservation.
